# Assessment of FBA Based Gene Essentiality Analysis in Cancer with a Fast Context-Specific Network Reconstruction Method

**DOI:** 10.1371/journal.pone.0154583

**Published:** 2016-05-04

**Authors:** Luis Tobalina, Jon Pey, Alberto Rezola, Francisco J. Planes

**Affiliations:** CEIT and Tecnun (University of Navarra), Manuel de Lardizábal 15, 20018, San Sebastian, Spain; University of Erlangen-Nuremberg, GERMANY

## Abstract

**Motivation:**

Gene Essentiality Analysis based on Flux Balance Analysis (FBA-based GEA) is a promising tool for the identification of novel metabolic therapeutic targets in cancer. The reconstruction of cancer-specific metabolic networks, typically based on gene expression data, constitutes a sensible step in this approach. However, to our knowledge, no extensive assessment on the influence of the reconstruction process on the obtained results has been carried out to date.

**Results:**

In this article, we aim to study context-specific networks and their FBA-based GEA results for the identification of cancer-specific metabolic essential genes. To that end, we used gene expression datasets from the Cancer Cell Line Encyclopedia (CCLE), evaluating the results obtained in 174 cancer cell lines. In order to more clearly observe the effect of cancer-specific expression data, we did the same analysis using randomly generated expression patterns. Our computational analysis showed some essential genes that are fairly common in the reconstructions derived from both gene expression and randomly generated data. However, though of limited size, we also found a subset of essential genes that are very rare in the randomly generated networks, while recurrent in the sample derived networks, and, thus, would presumably constitute relevant drug targets for further analysis. In addition, we compare the *in-silico* results to high-throughput gene silencing experiments from Project Achilles with conflicting results, which leads us to raise several questions, particularly the strong influence of the selected biomass reaction on the obtained results. Notwithstanding, using previous literature in cancer research, we evaluated the most relevant of our targets in three different cancer cell lines, two derived from Gliobastoma Multiforme and one from Non-Small Cell Lung Cancer, finding that some of the predictions are in the right track.

## Introduction

Recent findings show that cancer cells adapt their metabolic processes to enhance proliferation [1,2]. To that end, cancer cells consume additional nutrients and divert those nutrients into macromolecular synthesis pathways. Apart from alterations in glucose metabolism, the so called Warburg effect, more have been reported in the synthesis of nucleotides, amino acids and lipids [3,4]. In addition, relevant mutations in metabolic genes and accumulations of key metabolites have been detected in cancer cells [5]. In light of these evidences, the study of cellular metabolism in cancer research has been actively reawakened. Holistic systems biology approaches, based on genome-scale metabolic networks and high-throughput “omics” data, open new avenues to exploit metabolic disorders of tumour cells, particularly for addressing different unmet clinical needs in cancer.

Different methods exist to analyze genome-scale metabolic networks of human cancer cells. Constraint-based modeling (CBM) is an emergent area in Systems Biology that includes an increasing set of methods [6,7]. The most prominent method in CBM is Flux Balance Analysis (FBA), which assumes that the fluxes in the network follow a biological objective function to be optimized, typically cellular growth [8]. Growth is modelled here as an additional artificial reaction involving the metabolic requirements, in terms of building blocks and energy, for producing a gram dry weight (gDW) of biomass. FBA allows us to conduct gene essentiality analysis (GEA) at the metabolic level, namely by identifying those genes whose individual deletion prevent growth reaction from being active [9]. Synthetic lethality, which refers to two (or more) non-essential genes whose simultaneous deletion becomes lethal for a given phenotype, can be similarly accomplished. Importantly, the first application of FBA-based GEA to human metabolism and cancer research was accomplished in [10]. They revealed that haem oxygenase is synthetically lethal with the tumour suppressor fumarate hydratase. This result was later experimentally validated, showing its relevance to treat leiomyomatosis and renal-cell cancer, as germline mutations of fumarate hydratase underlie this cancer [11]. This successful result showed that FBA-based GEA is a suitable approach to elucidate novel drug targets in cancer.

FBA-based GEA starts from a reference genome-scale metabolic network of human metabolism, such as Recon2 [12]. In order to capture cancer-specific metabolic features, this reference network must be contextualized with available experimental data [13]. The manual process of building a reliable context-specific metabolic network is complex and time consuming [14]. For this reason, automatic network reconstruction algorithms have been proposed, typically based on gene/protein expression data. Given the wealth of transcriptomic data, mRNA expression data is the most frequent type of data used in the different reconstruction methods. A non-exhaustive list of this type of methods includes: GIMME [13], iMAT [15], E-Flux [16], MBA [17], PROM [18], MADE [19], INIT [20], or MIRAGE [21].

The results obtained from FBA-based GEA are dependent on the different elements involved in this network reconstruction process, i.e. reference network, defined growth medium, gene expression data and reconstruction algorithm. However, to our knowledge, no extensive assessment evaluating the influence of the metabolic reconstruction process and expression data on the results of gene essentiality analysis has been carried out to date in cancer. To that end, in this article, we conducted an extensive study for different types of cancers from the Cancer Cell Line Encyclopedia (CCLE) [22] so as to disentangle the effect of some of these factors in the resulting list of essential genes. In order to more clearly observe the effect of cancer-specific expression data, we did the same analysis using randomly generated expression patterns. In addition, we used high-throughput gene silencing data [23] to extensively test the predictions of the FBA-based GEA approach. Finally, we contrasted literature data about predicted essential genes in three cancer cell lines: two derived from Gliobastome Multiforme (GBM) and one from Non-Small Cell Lung Cancer (NSCLC).

To be able to conduct this extensive study, we introduce a fast network reconstruction algorithm based on gene expression data, which is treated using the Gene Expression Barcode [24], a robust statistical method developed to predict expressed and non-expressed genes in microarrays.

## Methods

### Network reconstruction model

Network reconstruction algorithms address the problem starting with a group of reactions that should be present based on previous experimental evidence, typically gene/protein expression levels. These reactions do not usually form a coherent network [25]. Indeed, they are not necessarily connected to each other, may form separated clusters or even be isolated from the rest. Thus, reconstruction algorithms fill in the gaps until a coherent network is obtained. Hypothesized reactions come from a database of known biochemical reactions, generally associated with the organism under study. In addition, note that it is also typical to avoid some reactions in the reconstruction because of experimental evidence of their absence [15].

Current reconstruction algorithms typically rely on Mixed Integer Linear Programming (MILP). We, instead, make use of an iterative strategy based on linear programs (LP), as MILP formulations are not sufficiently fast for the intended study. It is also the case that each reconstruction algorithm is usually focused towards the integration of a different type of one or more input experimental information. Because of this, in most cases, the results obtained from each one of them are not easily comparable. In our case, we focus on the use of mRNA transcript level data, as this is the most easily accessible data source in cancer. As detailed below, we used the Gene Expression Barcode [24], an elegant technique to select expressed and non-expressed genes, which eventually constitutes the source of evidence to contextualize metabolic processes.

Another feature of our reconstruction algorithm is that it supplies networks directly amenable to FBA, as we will carry out Gene Essentiality Analysis based on FBA in our study. This means that the reconstructed network should be able to produce biomass while it fulfills the steady state condition. Most other reconstruction algorithms are designed to guarantee the later but not the former.

Our algorithm distinguishes from others in several ways, apart from the fact that most of them rely on MILP formulations. GIMME [13] and iMAT [15] also use mRNA transcript level information, but its treatment is less elaborated than the one conducted with Barcode. MADE makes use of differential expression [19], focusing on metabolic adaptation between at least two scenarios. INIT is geared towards the use of more than one type of data [20]. MBA requires the definition of a core set of reactions forced to be included in the reconstruction [17]; however, while defining this active core is possible for well-known tissues, this is questionable when the available evidence is limited to gene expression data, typically involving conflicts between expressed and non-expressed genes and reactions due to post-transcriptional regulatory events [15,26]. MIRAGE extends upon MBA accounting, among other things, for biomass production [21]. PROM [18] and E-Flux [16] belong to a different family of methods, where maximum allowable fluxes are adjusted using gene expression data. In particular, PROM integrates metabolism with regulatory networks, requiring a large gene expression dataset with genetic and environmental perturbations.

Conceptually, our algorithm takes an approach that is more similar to iMAT than to other algorithms. Both classify reactions into high (*H*), moderate (*M*) and low (*L*) activity based on gene expression data and try to balance the inclusion of *H* and *L* reactions using the objective function. Unlike iMAT, we also minimize *M* reactions to some extent, so as to obtain a minimal network that satisfies the set of constraints. In addition, our algorithm adds the requirement of biomass production, as it is intended for obtaining networks directly amenable to FBA. However, as noted above, the main contribution of our approach with respect to iMAT is a significant reduction of computation time, while respecting the quality of the solution.

For the reduction of the computation time, our algorithm goes in the same direction as the algorithm recently presented in [27], termed FastCore. This algorithm uses a multistep approach based on linear programming, but it is conceptually similar to MBA, as it also forces the inclusion of a core set of reactions. Apart from the way it handles the inclusion of reactions, which is based on a three level classification from gene expression data, our algorithm also differs from FastCore in that it uses the concept of reduced cost from linear programming theory to guide the iterative solution process. In addition, we take into account the effects of different stoichiometric representations [28] by formulating the problem with respect to the maximum allowable flux through each reaction as given by a Flux Variability Analysis (FVA) [29].

Overall, our approach has been designed with the specific needs of this study in mind. A simplified version of our algorithm is presented below. Full technical details of our approach can be found in S1 Text.

### Overview of our linear programming-based algorithm

Consider a general metabolic network with *C* compounds and *R* reactions represented by its stoichiometric matrix *S* [30]. We denote *Irr* the set of irreversible reactions. For convenience, each reversible reaction contributes two different irreversible reactions to the total number *R*. These two irreversible reactions are denoted *f* and *b*, forward and backward, respectively, each of which represents the original reversible reaction in one different direction [31]. The set of forward and backward steps that arise from reversible reactions are denoted *Rev*.

The flux through each reaction *i* (*i* = 1,…,*R*) is represented by a continuous variable *v*_*i*_. After the split of reversible reactions, fluxes can only take non-negative values, bounded by a maximum flux value, $v_{i}^{\text{max}}$ (Eq 1). To later apply FBA-based GEA, we also enforce the steady state condition (Eq 2) and a minimum flux $v_{biomass}^{*}$ through the biomass reaction (Eq 3). For those compounds taken from or excreted to the medium, exchange reactions were added appropriately.

$$0\leq v_{i}\leq v_{i}^{\text{max}}\;i=1,\ldots ,R$$ (1)

$$\sum_{i=1}^{R}S_{ci}\cdot v_{i}=0\;\forall c\in C$$ (2)

$$v_{biomass}\geq v_{biomass}^{*}$$ (3)

To properly define $v_{i}^{\text{max}}$ for each reaction, we perform a Flux Variability Analysis (FVA) [29] under constraints (1)-(3). Uptake reaction bounds from the growth-medium under consideration are included in Eq 1.

We also define a continuous variable *z*_*i*_ for each reaction, bounded between 0 and 1 (Eq 4), which may force a minimum flux through its associated reaction, *v*_*i*_ (Eq 5). δ is a strictly positive constant with a maximum value of 1 that fixes the lower bound on *v*_*i*_ in relation with the value of *z*_*i*_ with respect to $v_{i}^{\text{max}}$. The inclusion of $v_{i}^{\text{max}}$ in Eq 5 as calculated by FVA allows us to set an activation threshold independent of the stoichiometric representation. We remark that this set of variables is continuous, as in [27], and not binary, as in a number of previous works [15,17].

$$0\leq z_{i}\leq 1\;i=1,\ldots R$$ (4)

$$\delta \cdot v_{i}^{\text{max}}\cdot z_{i}\leq v_{i}\;i=1,\ldots ,R,\;0<\delta \leq 1$$ (5)

Our objective is to minimize the number of reactions in *L* while maximizing those in *H*. For that, our objective function minimizes the sum of fluxes through reactions belonging to *L* with a weight *W*^*L*^, as well as the flux through reactions in *M* with a weight *W*^*M*^, while maximizing the number of reactions in *H* using *z* variables with a weight *W*^*H*^ (Eq 6). The term δ⋅$v_{i}^{\text{max}}$ in Eq 6 allows us to avoid the flux bias introduced by the specific stoichiometric representation of reactions. Different criteria to establish these weights are discussed in the Results section.

$$\text{min}\;W^{L}\sum_{i=1|i\in L}^{R}\frac{v_{i}}{\delta \cdot v_{i}^{\text{max}}}+W^{M}\sum_{i=1|i\in M}^{R}\frac{v_{i}}{\delta \cdot v_{i}^{\text{max}}}-W^{H}\sum_{i=1|i\in H}^{R}z_{i}$$ (6)

As noted above, it is common to set *z*_*i*_ as a binary variable, but relaxing that constraint, as done here, achieves the same “flux diversification” effect desired [27]. Minimizing the sum of fluxes for *L* and *M* is not the same as minimizing the number of reactions in *L* and *M*, but it allows us a linear formulation of the problem without negatively influencing the final solution in terms of quality. Overall, with these features, we avoid a mixed binary formulation, harder to solve because of the integrality constraints on some of the variables [32].

Since we have split the reversible reactions into two irreversible steps, but have added no constraint guaranteeing that only one of them is active at a time, solving this problem (Eq 6 subject to Eqs 1–5) will give us a solution where all forward and backward steps from reversible reactions in *H* are active, even if their net flux (*v*_*f*_−*v*_*b*_) is zero. Note that this does not occur with reversible reactions in *L* or *M*, because minimizing the sum of fluxes already enforces the usage of reversible reactions, if necessary, only in one direction.

**Fig 1:**
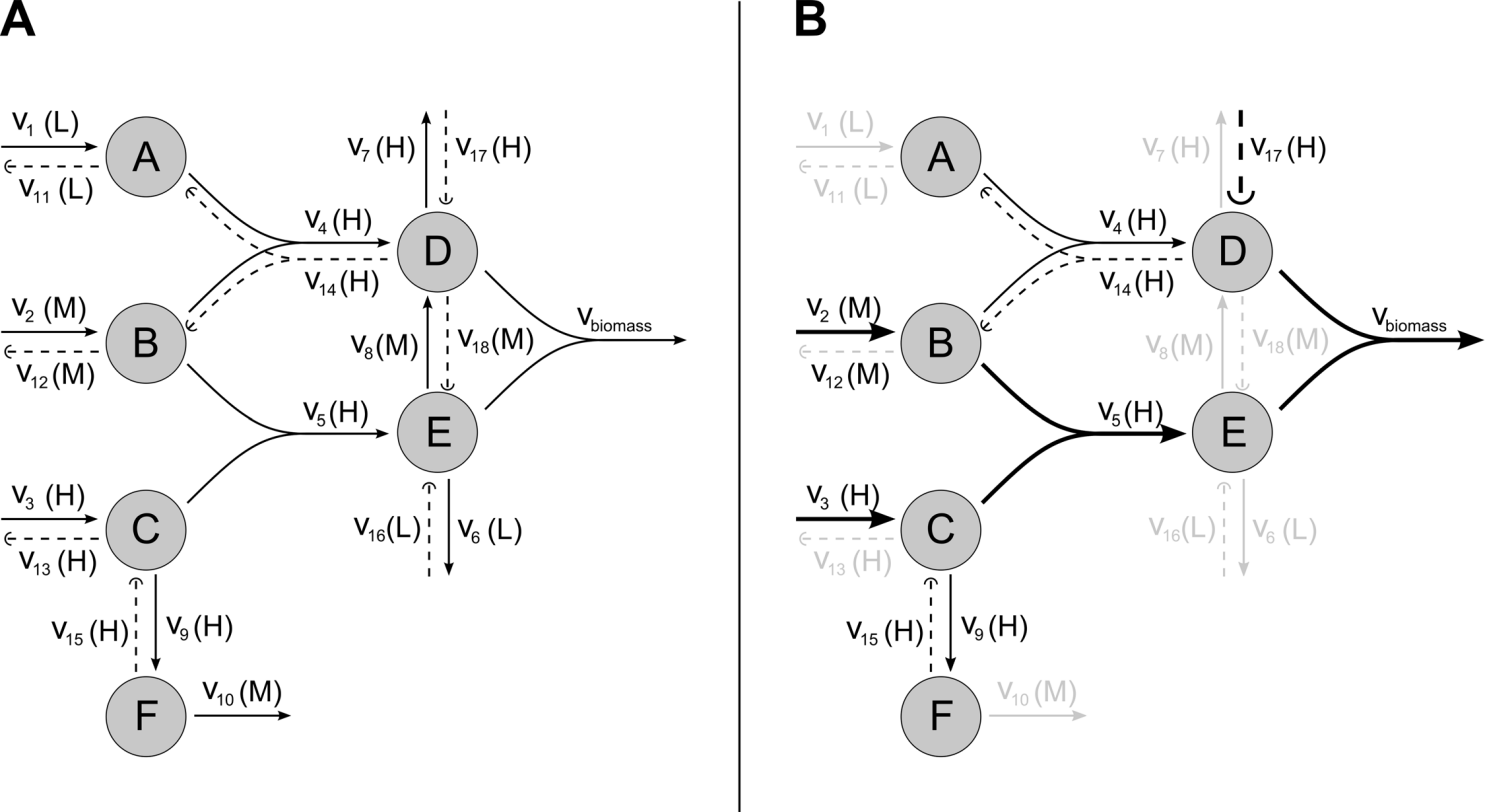
Illustration of the issue of spurious cycles of reversible reactions from the *H* set appearing in our linear programming formulation. A) Example reference metabolic network with a 3-level classification of reactions. It involves ten reactions plus the biomass reaction. Reversible fluxes are split into two non-negative steps. Backward reactions are shown in dashed line. Reactions 3, 4, 5, 7 and 9 are classified as *H*; reactions 2, 8 and 10 as *M*; and reactions 1 and 6 as *L*. B) Solution obtained when solving the linear program defined by Eq 6 subject to Eqs 1–5. Thicker arcs represent active reactions, cycles involving the forward and backward steps of a reversible reaction in *H* are represented with thinner lines and inactive reactions are colored in light grey.

This issue is illustrated in Fig 1. Fig 1A shows an example reference metabolic network, including the classification of reactions as *H*, *M* or *L*. Fig 1B shows the resulting solution once the linear program defined by Eq 6 subject to Eqs 1–5 is solved. It can be observed that the solution certainly produces biomass via reactions 2 (*M*), 3 (*H*), 5 (*H*) and 17 (*H*). In addition, it activates two cycles with net flux equal to zero, namely the first one involving reactions 4 (*H*) and 14 (*H*) and the second one involving reactions 9 (*H*) and 15 (*H*). The presence of these spurious cycles is a consequence of the non-binary formulation proposed above, which requires an iterative procedure that disentangles whether (or not) these reversible reactions in *H* can be included in the reconstruction in combination with other reactions.

The iterative procedure we used is described in detail in S1 Text. It is based on linear programming and it makes use of the concept of reduced cost (taken from linear programming theory) to guide and accelerate the iterative solution process.

### Reaction classification

The input of the reconstruction algorithm is the reaction classification as highly (*H*), medium (*M*) or lowly (*L*) expressed. This information is obtained from gene expression experiments, in our case collected from GEO database [33].

We focused on Affymetrix HGU133plus2 arrays, which can be processed using Barcode [24]. This method is designed to be able to work with just one sample and make it comparable to others, instead of needing several samples at the same time. We preprocessed the data using Barcode’s R script, using one sample at a time. We retrieved the Z-score values obtained from this algorithm, which is equivalent to processing each sample with fRMA [34].

Because the Z-scores retrieved from Barcode were given at the probe-set level, using gene-probe relationships annotated in hgu133plus2.db R package, we obtained the gene Z-score value as the median value of the corresponding Z-scores of its associated probe-sets. Each gene value was transformed into present(1)/absent(0) call using Barcode’s criteria. Present genes are classified as high (+1) and absent genes as low (-1).

Finally, reactions are classified as highly, medium or lowly expressed using gene-protein-reaction rules and the gene expression classification mentioned above [35] (see S1 Text for a more detailed explanation). Those reactions for which no gene expression is available or that are not related to any gene (e.g. spontaneous reactions) are classified as medium expressed.

### Gene Essentiality Analysis

Essential genes are defined here as those genes whose removal render the cell unable to produce biomass. Using the Boolean gene-protein-reaction rules incorporated in genome-scale metabolic networks such as Recon2 [12], we can evaluate which reactions will stop working after a particular gene is deleted. Thus, a gene knock-out is simulated by setting the upper and lower bounds of the corresponding reactions to zero in an FBA calculation, and checking whether (or not) the remaining network is still able to produce biomass.

In order to reduce the number of FBA calculations required to check the essentiality of every single gene, we first calculated the maximum biomass possible in the wild-type network and searched for a flux distribution with minimum sum of fluxes through reactions for which gene-to-reaction mapping is defined. If a particular gene knock-out does not affect any reaction in that optimal flux distribution, we can be certain that a new FBA calculation will give us the same solution as in the wild-type network and we can therefore skip such gene knockout.

### Comparison to experimental data

In order to assess the accuracy of our approach to predict essential genes, we used high-throughput silencing experiments taken from project Achilles [23]. We derived a score for each gene in each cell line following the method introduced in [36]. However, we multiplied the obtained scores by -1 so that the lower the score, the more essential the gene is supposed to be, as it happens with the shRNA fold changes in the high-throughput silencing experiments. We then compared the distribution of the scores of the obtained essential metabolic genes versus the nonessential metabolic genes using a one-sided two-sample Kolmogorov-Smirnov test, as suggested in [10]. This test helps us to see if the obtained essential genes are biased towards lower, more essential scores. However, the bias may be significant but not sufficiently large so, in addition, we measured the proportion of obtained essential genes with a negative Aquilles-based score in each scenario, a point where the probability of the gene being essential is higher than being non-essential. In fact, we noticed that only a fraction of the metabolic genes had a negative score in the Achilles data, so we want to make sure that the calculated essential genes are enriched in them.

## Results

The approach presented above is first applied to reconstruct the metabolic network of 174 cancer cell lines using gene expression data obtained from the Cancer Cell Line Encyclopedia (CCLE) [22]. The choice of this subset of cell lines was made taking into account the available high-throughput gene silencing data from project Achilles [23] (S1 Table). The technical performance of our approach is evaluated and compared with iMAT, the most similar approach to the one introduced here (S1 Text). Next, we perform FBA-based GEA over these reconstructed networks and assess the frequency with which each essential gene would appear in a network reconstructed from random expression data. In addition, we compare the obtained results to high-throughput gene silencing experimental results [23]. Finally, we contrasted literature data about predicted essential genes in two GBM-derived and one NSCLC-derived cell lines.

To this end, we used the original human metabolic network Recon2 [12] as reference network (a similar analysis for Recon1 can be found in S1 Text). This network provides a biomass reaction, which is directly used in this study. The growth medium was RPMI1640, defined as in [10]. In addition, reactions were classified as highly, medium or lowly expressed using gene-protein-reaction rules and the gene expression classification described in Methods section.

The algorithm was implemented in Matlab, using Cplex optimization software to solve the corresponding linear programs. The computation time needed to solve a single reconstruction problem using the strategy described above is in the order of seconds, in par with the performance of Fastcore [27]. On the instances our method was applied, computation time is generally below 10 seconds on a 64 bit Intel Xeon E5-1620 v2 at 3.70 GHz (4 cores) and 16 GB of RAM. This sets our algorithm as substantially faster than iMAT, where the median time to obtain a solution was around 57 seconds (stopping with a 0.5% optimality gap).

### Model parameters and reconstruction

In our reconstruction algorithm we have several parameters that require being fixed. The most relevant parameters are the weights *W*^*H*^, *W*^*M*^ and *W*^*L*^, as there is a conflicting trade-off between reactions in *H* and *L*. In particular, the use of all reactions in *H* may involve a significant number of reactions in *L*; similarly, a minimum use of reactions in *L* may imply a limited use of reactions in *H*. In order to study this trade-off between reaction in *H* and *L*, we propose the schemas in Table 1, with α = 10^3^. Schema 1 gives more weight to the minimization of reactions in *L* over the maximization of reactions in *H*; Schema 2 provides equal weight, while Schema 3 is the opposite of Schema 1. Details and sensitivity analysis of α and other parameters fixed in our algorithm can be found in S1 Text. Main conclusions achieved were robust to changes of these parameters.

**Table 1:** Weighting schemas used in the reconstruction algorithm proposed.

Schema	*W*^*H*^	*W*^*M*^	*W*^*L*^
**1**	α	1	α^2^
**2**	α	1	α
**3**	α^2^	1	α

When classifying reactions from gene expression data, avoiding the inclusion of reactions in *L* as much as possible might be more meaningful than trying to force the presence of all reactions in *H*, as a high gene expression signal does not necessarily translate into a high enzymatic activity. However, the identification of non-expressed genes constitutes a more difficult task [37]. For this reason, an approach closer to Schema 3 has been typically preferred.

**Fig 2:**
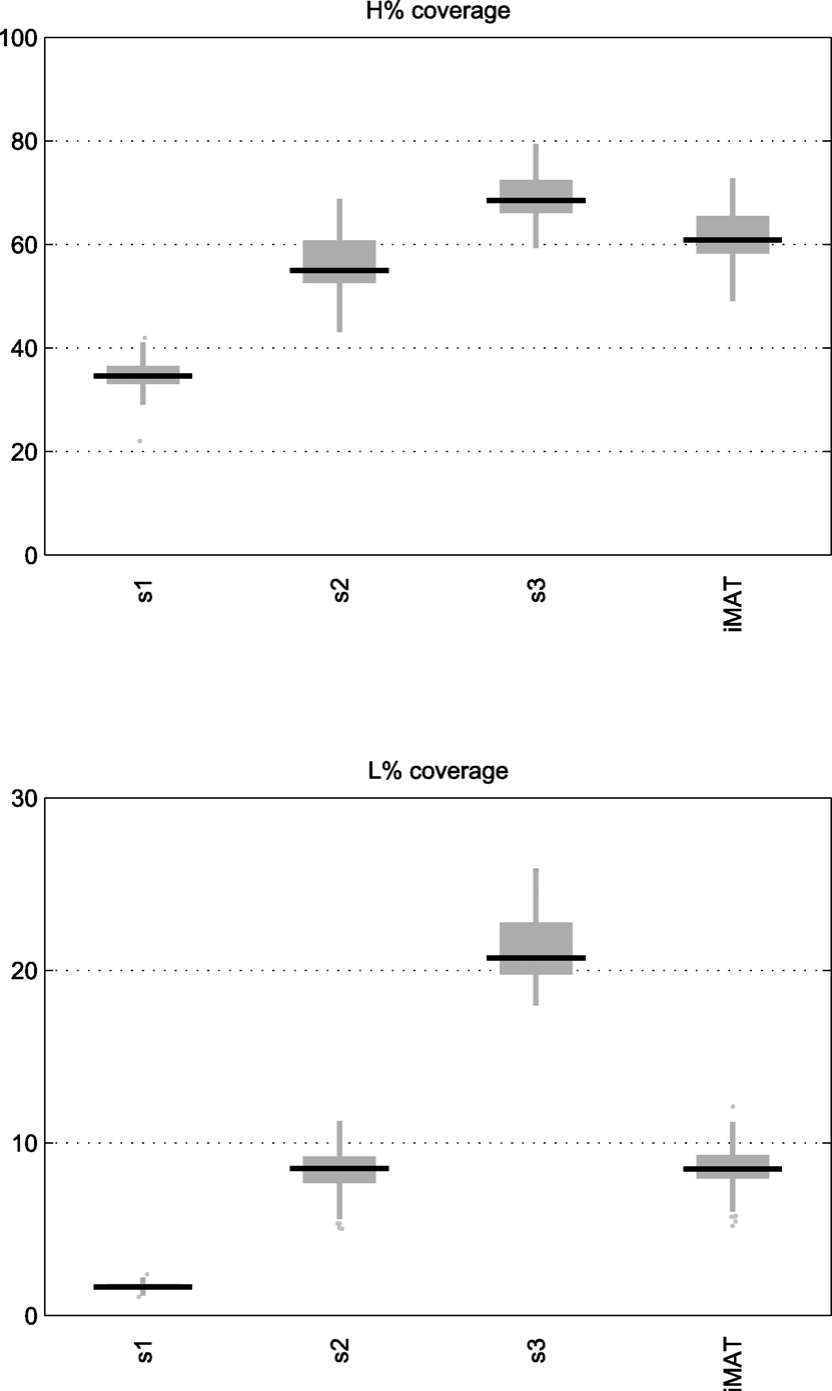
Boxplots of H and L reaction coverage in networks of selected cancer cell lines. Boxplots showing the percentage of H and L reactions included in the reconstructed context-specific networks of selected cancer cell lines using our algorithm under Schema 1, 2 and 3 and iMAT. The reference network used was Recon2.

We compared the performance of our reconstruction approach using the different schemas with iMAT. As can be seen in Fig 2, which shows the percentage of reactions classified as *H* and *L* that were included using each reconstruction algorithm, the avoidance of *L* reactions in Schema 1 has an impact on the number of reactions in *H* included in the model, providing a significantly different solution than Schema 3.

As expected, Schema 2 is the most similar to iMAT, as both provide equal weight to reactions in *H* and *L*. It can be observed that the number of L reactions included is very similar and the number of *H* reactions included by our algorithm is somewhat lower. Overall, both methods obtain similar reconstructions in terms of the number of *H* and *L* reactions they include. Thus, we consider our algorithm a valid tool for the task at hand. Note that the maximum possible percentage of *H* reactions included in the reconstruction does not necessarily reach 100% as there might be reactions that cannot operate in steady state under the imposed medium conditions.

### Gene essentiality analysis

With a fast reconstruction algorithm in our hands, we can address the question of the extent to which the set of essential genes is being affected by context-specific expression data. To further explore this issue, we permuted the metabolic gene expression classification of each sample 10 times and reconstructed the corresponding networks followed by the calculation of their corresponding essential genes, leading to a background of almost 2000 random results.

**Fig 3:**
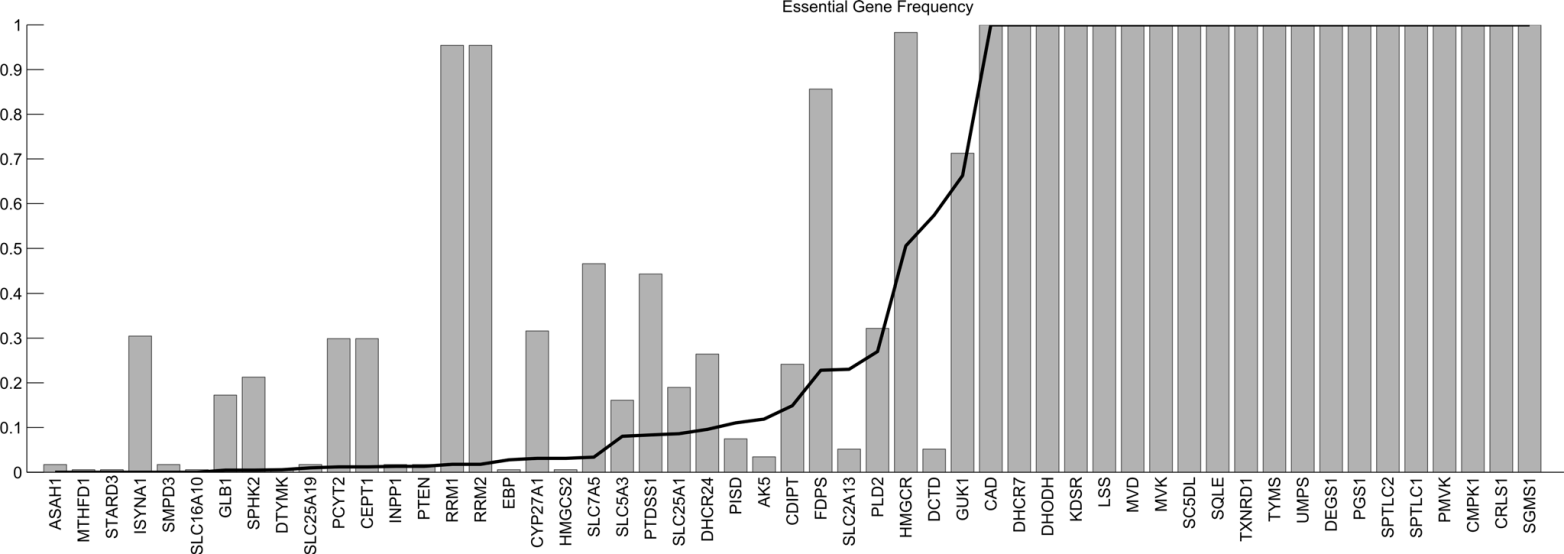
Essential gene frequency for reconstructed context-specific networks of selected cancer cell lines. Essential gene frequency for reconstructed context-specific networks of selected cancer cell lines using our algorithm with Schema 3 and Recon2 as the base network. The horizontal axis contains the ENTREZ Symbols of the obtained essential genes. The height of the bars indicates the fraction of samples in which the gene appears as essential. The height of the black line indicates the fraction of randomly reconstructed network in which the corresponding gene appears as essential.

Fig 3 shows the results of this experiment for Schema 3 (the list of genes and values can be found in S2 Table). As partially expected, there are some genes that are fairly common in any reconstructed network. The most extreme cases are genes that appear as essential whatever the input expression is. These are a direct consequence of the input reference network, the fixed growth medium conditions and the selected biomass reaction. This analysis confirms the extent to which these factors can affect the results.

Note that there also exist some essential genes very frequent in the individual samples but less frequent in the random networks. These would be, a priori, the most interesting ones, as they are more related than the other genes to the particular expression of the samples.

The most striking fact is that the list of obtained essential genes exclusive of each cancer type is fairly short. Only 6 genes appeared only in one cancer type when using our algorithm with Schema 3, 22 and 21 if we used Schema 1 and 2, respectively. We expected a more diverse set of essential genes for each cancer type.

Some previous work explored the essentiality concept under very diverse growth medium conditions [38] for some bacterial metabolic networks. They concluded the existence of a core set of reactions needed for biomass production independent of the selected growth medium. Our study leads to very similar insights for the case of network contextualization. The same conclusion was achieved for different parameter settings and scenarios, including the use of Recon1 and a general growth medium (see Table B in S1 Text).

### Comparison to high-throughput gene silencing experiments

A systematic effort to identify essential genes in different cancer cell types is being carried out in what is known as project Achilles [23]. The coverage of this project has grown during the last years [23,39,40]. They performed high-throughput gene silencing experiments to identify genetic vulnerabilities in different cell lines. Comparison of computationally obtained essential genes with the results coming from this type of experiments have been previously used to assess the validity of the approach [10].

**Fig 4:**
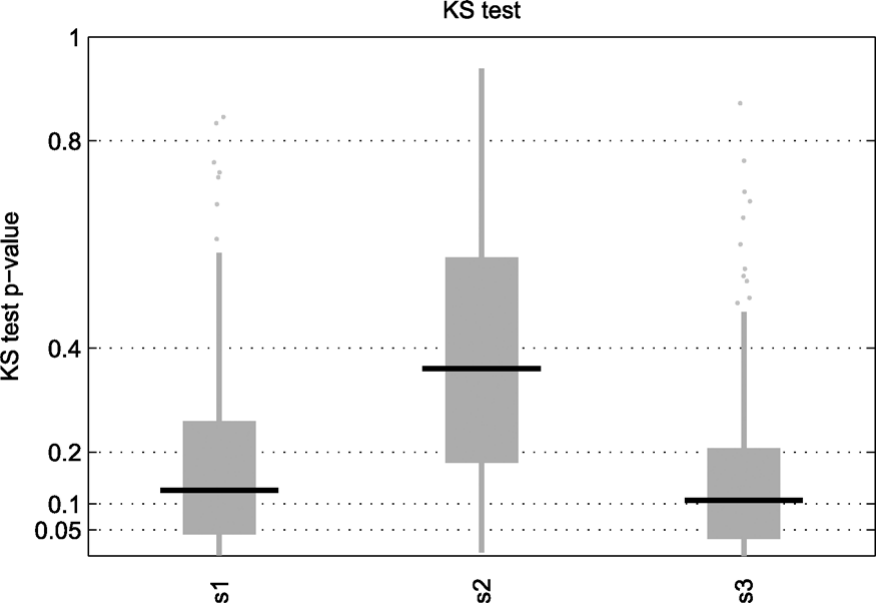
KS test p-value of essential genes in reconstructed context-specific networks. KS test p-value of essential genes obtained from the networks reconstructed from CCLE samples with matched Achilles experiment using our algorithm under Schema 1, 2 and 3. The base network is Recon2.

The results for the Achilles experiments are summarized per gene as explained in the Methods section, so that the more negative the score is, the more essential the gene is considered. Focusing on samples from the CCLE with matched Achilles experiments, we used a two-sample one-tailed Kolmogorov-Smirnov test between the metabolic essential genes obtained from our *in-silico* approach and the rest of metabolic genes to see if the former had generally lower scores than the later. Fig 4 shows the results for this analysis, where our algorithm (under different schemes) does not seem to obtain a high number of results below the 5% value (which should be later corrected for multiple hypothesis testing using, for example the false discovery rate (FDR)). S1 Text includes the same analysis as in Fig 4 but using Recon1, where results below the 5% value are more common (Fig I in S1 Text). However, we expected the FBA based GEA to give significant results with almost all the samples we tried it on. Note also that this analysis was recalculated for different parameter settings and we obtained similar results, though particularly inferior when a general growth medium was fixed (Table C in S1 Text).

**Fig 5:**
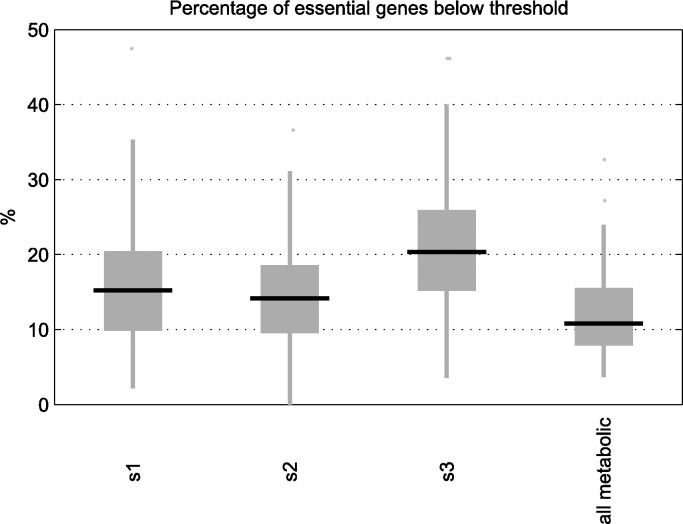
Percentage of essential genes satisfying Achilles-based threshold of essentiality. Percentage of essential genes obtained from the reconstructed context-specific networks of selected cancer cell lines using our algorithm under Schema 1, 2 and 3 that have a negative Achilles-based score, indicating a higher probability of being essential than non-essential according to processed gene silencing data obtained from project Achilles. The base network is Recon2. The last boxplot indicates this percentage when all the metabolic genes are taken into account.

In addition, we decided to count the fraction of genes with a negative Achilles-based score (i.e. those with a higher probability of being essential than non-essential), among the genes predicted as essential in our *in-silico* approach. We observed that the number is around 10 to 20%, regardless of the reconstruction method used (Fig 5). However, if we take all the metabolic genes represented in Recon2, we see that around 8 to 16% of them had a negative Achilles-based score. While [38] found that the core reactions in bacterial metabolic networks where enriched in experimentally proven essential genes, our results do not allow us to make the same claim. Note that when we replicated the same analysis for Recon1 (Fig J in S1 Text), in contrast with the results obtained in the KS test, we obtained very similar (even slightly worse) results than in Recon2. A similar conclusion was found for other parameter settings (Table C in S1 Text).

Knowing that some genes appear similarly in both random and cancer-specific expression based networks, while others are clearly tied to the cancer-specific expression pattern, we tried to identify a relationship between this and the corresponding experimental essentiality score. However, we were unable to find a significant correlation when focused on each particular CCLE sample, possibly due to the reduced sample size obtained as we restrict to essential genes that appeared only in less than a given threshold in the random samples. To overcome this issue, we merged the results of the CCLE sample set and analyzed whether, as this threshold was lowered, the fraction of genes with a negative Achilles-based score increases. This pattern was moderately found in the case of Schema 3 in both Recon1 and Recon2, but not in Schema 1 and 2 (S1 Text), which leaves inconclusive evidence for establishing a relationship between FBA predicted cancer-specific essential genes and Achilles data.

### Findings in the literature

Despite the results of our comparison with project Achilles data, we decided to take a look into the essential genes obtained for two GBM-derived cell lines (U251 and U87) and one NSCLC-derived cell line (A549). Since the data used in CCLE only considered a single sample for each cancer cell line, we applied the same approach to an additional set of samples obtained from GEO (see S1 Table).

We found interesting the case of the gene PLD2 (ENTREZ ID 5338). This gene appears as essential in 84% of the networks reconstructed from GBM samples with Schema 3, while it only appears in 27% of the networks reconstructed from random expression data and in 32% of the networks reconstructed from all the CCLE selected samples. This gene has been shown to play an important role in cancer [41] and to inhibit the proliferation of U251 cells when suppressed [42]. However, its Achilles-based score in the U251 cell line is 20.03 and 12.70 in the U87 cell line, which, in principle, would have not attracted our attention.

Other interesting genes are RRM1 (ENTREZ ID 6240), RRM2 (ENTREZ ID 6241), FDPS (ENTREZ ID 2224) and HMGCR (ENTREZ ID 3156), although these also appear as essential in many of the networks reconstructed from all the CCLE selected samples (but not in the randomly reconstructed networks). RRM1 and RRM2 form the enzyme ribonucleotide reductase, whose inhibition via GTI-2040 has been shown to have a potent antitumor activity in different cell lines, including U87 [43]. In the case of A549, siRNA-mediated silencing of RRM1 and RRM2 inhibited its growth [44]. On the other hand, FDPS inhibition has shown an increased paclitaxel-induced apoptotic cell death in U87 glioblastoma cells [45]. In addition, bisphosphonates have been found to inhibit FDPS activity [46], and there is research showing that they inhibit cell proliferation in A549 cells [47]. Finally, inhibition of HMGCR through simvastatin reduced cell growth in U87 cells [48] and increased apoptosis in an *in vivo* mouse GBM model [49]. Likewise, simvastatin has also been found to inhibit A549 proliferation [50], although it has also been proposed that it acts through Akt signaling dependent down-regulation of surviving [51].

The Achilles-based score for RRM1 in U251, U87 and A549 is -17.8360, -7.416 and 1.4952, respectively; 5.6092, 8.7197 and 7.3371 for RRM2; -4.08, 6.74 and -11.2250 for FDPS; 14.85, 5.16 and 11.7576 for HMGCR. Again, except for RRM1 in U251 and U87 and FDPS in U251 and A549, these genes would probably have not called our attention, but there is literature in their favour.

## Conclusion

In this work, we have evaluated the accuracy of the results obtained from Flux Balance Analysis based Gene Essentiality Analysis (FBA based GEA) when networks contextualized with gene expression are used. In order to carry out this research, we have introduced a new fast metabolic network reconstruction algorithm that has been used to evaluate the approach. We focused on the reconstruction of networks from samples in the Cancer Cell Line Encyclopedia (CCLE) that had an available high-throughput gene silencing experiment in project Achilles.

Current methods that apply gene essentiality analysis, to our knowledge, lack an assessment of their results against random input, which would allow us to distinguish them from those obtained because of algorithmic artifacts or the rigidity or incompleteness of the reference network. Here, we directly addressed this issue by evaluating the impact of cancer-specific gene expression in GEA in comparison with randomly generated expression patterns.

To achieve the objective above, the need of a fast reconstruction algorithm is a must. The reconstruction algorithm proposed here builds on the reasoning present in previous approaches [10,17,27]. Our approach is conceptually similar to iMAT [15], but, in line with FastCore [27], it provides us with network reconstructions in much less time, thanks to the iterative LP strategy used. This feature has allowed us to reconstruct a high number of networks in order to assess the frequency with which genes appear as essential in randomly generated data.

The results show that the resulting list of essential genes has a varying degree of dependence on the reference network, the reconstruction algorithm and context-specific expression data. Most relevant conclusions are discussed below.

First, we observed a clear role of the schema (parameters *W*^*H*^, *W*^*M*^ and *W*^*L*^) in the reconstruction, which illustrates the need to decide over the treatment of highly and lowly expressed reactions. Systematic procedures as Barcode [24], which was used in this article, are an essential part in this debate. Barcode, for example, is very restrictive for expressed genes and it is more likely to have false negatives. If that is the case, Schema 3 could be the preferred option, as it biases the reconstruction towards including reactions classified as *H*. However, Schema 1 would be the most realistic approach if we could identify non-expressed genes accurately.

Second, we emphasize the need of a robust gene essentiality analysis, as the general results seem insensitive to the reconstruction process, at least when transcriptomic data is exclusively used. In particular, using randomly generated expression patterns, we found essential genes that are more likely to appear than others, which illustrate a higher dependence on the reference network, fixed growth medium and the selected biomass reaction than on the context-specific expression data.

Third, we found essential genes that are rare in the randomly generated networks and recurrent in cancer samples. These can be considered as highly dependent on the context-specific expression data and are presumably the ones we would find more interesting at first glance. However, the comparison to experimental high-throughput silencing data cannot be considered to agree with the computational results, suggesting the need to consider additional factors.

We believe the selection of the appropriate biomass reaction, or an appropriate metabolic task function, is key in the whole process, as it plays a central part in the definition of essentiality. For illustration, we tried some random alterations to the biomass reaction (S1 Text) and, as expected, had an impact on the results. In fact, the selection of an appropriate biomass function is probably one of the biggest challenges ahead in the FBA based GEA methodology. The use of the biomass function is well founded in bacterial metabolism, but it may not be the best option for modeling some types of cancer. Furthermore, it is likely that this function will differ between different cell lines. More than a biomass reaction we will be probably looking for a cancer-specific metabolic task or objective function, as recently proposed in [52] and [53].

Another possibility is that the reference network (from which our reconstructions are derived) misses several reactions that would allow us to capture more differences at the reconstruction level. It is known that the central metabolism and some major pathways are well studied and represented in these networks, but a big part of the real metabolic possibilities of the cells might still be missing. It is also important to note that this approach does not consider in any way the cell rearrangement that may follow a knock-out intervention, which could provide an explanation for some false-positive predictions.

It should also be noted that high-throughput gene essentiality experiments are far from trivial and their analysis is also complicated. Hence, lists of essential genes based on the analysis of those experimental results would also likely need follow-up experiments to confirm them.

We would like to point out that focusing on the p-value of the KS test alone is not sufficient to test the relevance of the obtained list of essential genes. The two sample KS test is designed for comparing whether two observed sets may come from the same distribution or not. What we really want to check is if the obtained essential genes are ranked at the very top of the experimentally obtained rank of essential genes. If that happens, the KS test will give a significant p-value. However, if the KS test gives a significant p-value, it does not necessarily imply that the calculated genes fall at the very top of the list, they may just be biased, on average, towards the top. Hence, to complement the KS test, we calculated the proportion of obtained essential genes satisfying the essentiality threshold derived from the data in project Achilles. Under this measure, the list of obtained essential genes did not have a proportion very different from the one that can be obtained when focusing on all the metabolic genes. The same conclusion was found in the scenarios evaluated, including different reference networks (Recon1 and Recon2), growth media (RPMI and general growth medium) and reconstruction algorithm parameters.

Despite these discrepancies between the predicted genes and the Achilles data, we were able to find a number of genes in GBM with a higher frequency in the samples than in the random networks which have been shown to effectively reduce its proliferation when targeted [42,43]. We also found some additional genes in GBM and NSCLC with interesting references in the literature [44–51].

Constraint based modeling approaches have had a great success in the microbial metabolic modeling research. The success has been possible in part thanks to the great number of experiments and information that has been gathered in those organisms. As cancer cell lines are less well characterized than some microorganisms, data from projects like Achilles constitute a very valuable resource to boost the models’ performance. Until now, data on the real essentiality of metabolic genes was scarce. Although high-throughput silencing experiment data is complex and may contain inaccuracies, it is our best chance to improve these models.

Overall, we believe that constraint based approaches hold great promise in the study of cancer metabolism. Notwithstanding, for its application towards the identification of essential genes, the technique needs to carefully consider the assumptions it makes and identify the correct formulation of the question at hand. Our framework exposes these issues and can be a key component to solve the final puzzle.

## Supporting Information

S1 Code fastReconstructionAlgorithm. (ZIP)

S1 Table List of samples. (XLS)

S2 Table FBA based GEA results for Schema 3. (XLS)

S1 Text Supplementary methods and results. (PDF)
