# Supplementary material for: Assessment of FBA Based Gene Essentiality Analysis in Cancer with a Fast Context-Specific Network Reconstruction Method
Source: PLoS One. 2016 May 4;11(5):e0154583. doi: 10.1371/journal.pone.0154583 (PMC4856428; doi:10.1371/journal.pone.0154583)
Supplement: S1 Text — (PDF) [file pone.0154583.s004.pdf]

Assessment of FBA Based Gene Essentiality Analysis in Cancer with a Fast Context-specific Network Reconstruction Method

Luis Tobalina[1], Jon Pey[1], Alberto Rezola[1] and Francisco J. Planes[1,*]

[1] CEIT and Tecnun (University of Navarra), Manuel de Lardizábal 15, 20018 San Sebastian, Spain

1 SUPPLEMENTARY METHODS

1.1 Network reconstruction model

We present below a multistep strategy based on a linear programming formulation to reconstruct a metabolic network with gene expression data. The different steps of our reconstruction algorithm are detailed below.

1.1.1. Basic network

Consider a general metabolic network with C compounds and R reactions represented by its stoichiometric matrix S [1]. We denote *Irr* the set of irreversible reactions. For convenience, each reversible reaction contributes two different irreversible reactions to the total number R. These two irreversible reactions are denoted f and b, forward and backward, respectively, each of which represents the original reversible reaction in one different direction [2]. The set of forward and backward steps that arise from reversible reactions are denoted *Rev*.

The flux through each reaction i (i=1,…,R) is represented by a continuous variable v_i. After the split of reversible reactions, fluxes can only take non-negative values, bounded by a maximum flux value, $v_i^{\max}$ (Eq. 1). To later apply FBA-based GEA, we also enforce the steady state condition (Eq. 2) and a minimum flux $v_{biomass}^*$ through the biomass reaction (Eq. 3). For those compounds taken from or excreted to the medium, exchange reactions were added appropriately.

$$0 \le v_i \le v_i^{\max} \qquad i = 1,\dots,R \qquad (1)$$

$$\sum_{i=1}^{R} S_{ci} \cdot v_i = 0 \qquad \forall c \in C \qquad (2)$$

$$v_{biomass} \ge v_{biomass}^* \qquad (3)$$

To properly define $v_i^{\max}$ for each reaction, we perform a Flux Variability Analysis (FVA) [3] under constraints (1)-(3). Uptake reaction bounds from the growth-medium under consideration are included in Eq. 1. We also limit the maximum flux through any reaction in the network for which no bound is given to 1000 before applying FVA in order to avoid unbounded fluxes.

We also define a continuous variable z_i for each reaction, bounded between 0 and 1 (Eq. 4), which may force a minimum flux through its associated reaction, v_i (Eq. 5). δ is a strictly positive constant with a maximum value of 1 that fixes the lower bound on v_i in relation with the value of z_i with respect to $v_i^{\max}$. The inclusion of $v_i^{\max}$ in Eq. 5 as calculated by FVA allows us to set an activation threshold independent of the stoichiometric representation. We remark that this set of variables is continuous, as in [4], and not binary, as in a number of previous works [5,6].

$$0 \le z_i \le 1 \qquad i = 1,\dots R \qquad (4)$$

$$\delta \cdot v_i^{\max} \cdot z_i \le v_i \qquad i = 1,\dots,R \quad 0 < \delta \le 1 \qquad (5)$$

Our objective is to minimize the number of reactions in L while maximizing those in H. For that, our objective function minimizes the sum of fluxes through reactions belonging to L with a weight W^L, as well as the flux through reactions in M with a weight W^M, while maximizing the number of reactions in H using z variables with a weight W^H (Eq. 6). The term $\delta \cdot v_i^{\max}$ in Eq.6 allows us to avoid the flux bias introduced by the specific stoichiometric representation of reactions. Note here that blocked reactions ($v_i^{\max} = 0$) are removed and can be left out of Eq. 6. Different criteria to establish these weights are discussed in the Results section of the main paper.

$$\min W^L \sum_{i=1|i\in L}^{R} \frac{v_i}{\delta \cdot v_i^{\max}} + W^M \sum_{i=1|i\in M}^{R} \frac{v_i}{\delta \cdot v_i^{\max}} - W^H \sum_{i=1|i\in H}^{R} z_i \qquad (6)$$

As noted above, it is common to set z_i as a binary variable, but relaxing that constraint, as done here, achieves the same "flux diversification" effect desired [4]. Minimizing the sum of fluxes for L and M is not the same as minimizing the number of reactions in L and M, but it allows us a linear formulation of the problem without negatively influencing the final solution in terms of quality. Overall, with these features, we avoid a mixed binary formulation, harder to solve because of the integrality constraints on some of the variables [7].

Since we have split the reversible reactions into two irreversible steps, but have added no constraint guaranteeing that only one of them is active at a time, solving this problem (Eq.6 subject to Eq.1-Eq.5) will give us a solution where all forward and backward steps from reversible reactions in H are active, even if their net flux ($v_f - v_b$) is zero. Note that this does not occur with reversible

reactions in L or M, because minimizing the sum of fluxes already enforces the usage of reversible reactions, if necessary, only in one direction. For this reason, we need an iterative procedure that disentangles whether these reversible reactions in H can certainly be included in the reconstructed network.

On the other hand, the solution resulting from this step directly provides us with the subset of irreversible reactions from H that will be involved in our final reconstruction. For this reason, the flux of irreversible reactions from H that have not been activated in this first step is set to zero for the rest of the iterative process (Eq. 7).

$$v_i = 0 \quad \forall i | i \in H, i \in Irr, i \notin D \qquad (7)$$

Overall, this first step provides a first draft network D that will be expanded in the next steps. Reversible reactions in H with net flux equal to zero cannot be directly included in D and require further analysis to evaluate their presence in the final reconstruction.

1.1.2. Iterative process for reversible reactions in *H*

The aim of this iterative process is to determine which reversible reactions in H will be part of the final reconstructed network, in particular those with net flux equal to zero in the previous step. During the iterative process, we will gradually increment the number of reactions included in our draft network D. In each iteration, we will set the penalty W^L and W^M of those reactions already included in the solution in previous iterations to zero, as once a reaction is included in the draft, there is no need to penalize it further. Similarly, we will set the W^H bonus of reversible reactions in H already included in the draft from previous iterations to zero. Note that the W^H bonus of irreversible reactions in H is kept to guide the addition of reactions in D. These variations lead to a new objective function, which is represented by Eq. (8).

$$\min W^L \sum_{i=1|i\in L, i\notin D}^{R} \frac{v_i}{\delta \cdot v_i^{\max}} + W^M \sum_{i=1|i\in M, i\notin D}^{R} \frac{v_i}{\delta \cdot v_i^{\max}} - W^H \sum_{i=1|i\in H, i\in Irr}^{R} z_i - W^H \sum_{i=1|i\in H, i\in \mathrm{Re}v, i\notin D}^{R} z_i \qquad (8)$$

In order to evaluate whether a reversible reaction from H, currently not included in D, must be added into the reconstruction, we need to solve the linear program defined by Eq. 8 s.t. Eq. 1-5,7, in two different scenarios: one with flux equal zero in the forward direction, $v_f = 0, f \in H, f \in \mathrm{Re}v, f \notin D$, and the other with flux equal zero in the backward direction, $v_b = 0, b \in H, b \in \mathrm{Re}v, b \notin D$. If $v_b > 0$ in the first scenario or/and $v_f > 0$ in the second scenario, then this reversible reaction takes part in the final reconstruction, as well as additional reactions from the sets H, M and L required to perform in steady state. We may also need to add other reversible reactions from H currently not in D and, therefore, their analysis will not be further required. In case that $v_b = 0$ in the first scenario and $v_f = 0$ in the second scenario, this reaction is discarded from the final reconstruction. We will refer to this process as **Iteration A**.

The strategy described above, though general, may require a large number of linear programs, as we need to individually check each reversible reaction. In order to reduce computation time, we introduce an intermediate algorithmic step, based on the concept of reduced cost from linear programming, which allows us to minimize the number of linear programs to be solved. Full details are provided below.

1.1.3. Efficient implementation of iterative process for reversible reactions in *H*

If we knew in which direction was going to work each reversible reaction in a possible solution, we could block the reactions in the opposite direction and solve the previous problem to recover that solution. However, as this is not the case, we will make a guess and then use linear programming theory to further improve it.

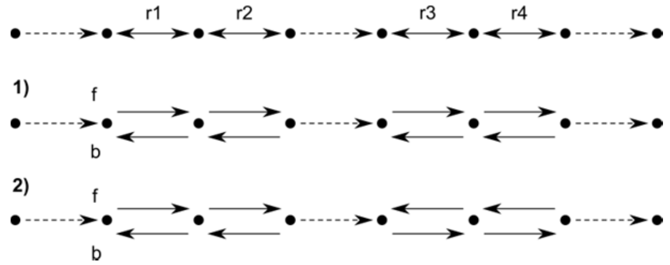

Figure A. Toy example summarizing key steps in our algorithm. The series of reactions in this figure contains some reactions classified as medium (discontinuous arrows) and some as high (continuous arrows). Reactions classified as high (r1, r2, r3 and r4) are defined as reversible. Two possibilities to split the reversible reactions into a pair of irreversible steps are pictured in Figure A1 and A2. Note that in Figure A2, forward direction for reactions r3 and r4 have been chosen the opposite as in Figure A1. The application of Iteration B would activate all the reactions in Figure A2 if the couple (r1, r2) or (r3, r4) are assigned to the *J* set. If that is not the case, we would need to check each reversible reaction individually using Iteration A.

We will first solve the linear program defined by Eq. (8) subject to Eq. (1)-(5), (7), in two different scenarios. In particular, for all the reversible reactions in H not included in D, we will set their fluxes to zero in one direction first ($v_f = 0 \quad \forall f \in H, f \in \mathrm{Re}v, i \notin D$) and later in the other ($v_b = 0 \quad \forall b \in H, b \in \mathrm{Re}v, b \notin D$), similarly to what is done in the FastCC algorithm in [4]. In addition, we will relax the bounds in the other direction, as observed in Eq. (9). The solution to this linear program may provide new reactions to the draft network D (see Figure A1), but the solution might have been better if we had selected some reversible reactions in the opposite direction.

$$v_f = 0, -ub_f \le v_b \le ub_b \quad \forall (f,b) \in H, \forall (f,b) \in \mathrm{Re}v, \forall (f,b) \notin D \qquad (9)$$

Here, we can make use of linear programming theory to improve upon our solution and eventually reduce the number of reactions that will need to be checked individually. Specifically, we will make use of the concept of reduced cost of a variable. This value indicates how much the objective value would theoretically change if we modify the value of a variable by one unit.

It is important to clarify that, for the determination of reduced costs, it was assumed that the proposed linear problem is solved handling the bounds on variables implicitly. In fact, most available linear programming solvers implicitly handle variable bounds, meaning that those bound constraints are not explicitly added to the constraint matrix. Under these circumstances, the non-basic variables are no longer necessarily zero and their reduced cost can take any real value.

For readers unfamiliar with linear programming, variables are classified as basic or non-basic. Non-basic variables are independent variables and their value is set equal to their upper or their lower bound. By contrast, basic variables are dependent variables and their value is obtained by solving the corresponding system of equations [7].

In the optimal solution, reduced cost of basic variables is zero, while it is usually nonzero for non-basic variables, unless the problem has alternative optimal solutions, where non-basic variables may have a reduced cost of zero. For a minimization problem, the reduced cost of non-basic variables at their lower bound will be positive, and for non-basic variables at their upper bound, it will be negative. These reduced costs can also be interpreted as the shadow prices of the lower and upper bound constraints, respectively, on these variables.

With the solution to our modified problem in our hand, we set the focus on the reduced costs of z_b variables associated to v_b variables, for which we have relaxed the lower bounds. If their reduced cost is positive, a decrease in their value implies a reduction in the objective function value. In this case, if we allow a small negative value for z_b, the corresponding v_b would be able to take a negative value (see Eq.(9)). This may be sufficient to activate another reaction from *H*, allowing another *z* variable to take a positive value and, thus, improving the objective function value. If their reduced cost is zero and they are non-basic variables, we have alternative optimal solutions, implying that a small change in the lower bound of that variable could lead to a different optimal solution and, therefore, we also need to look at these variables.

These backward reactions *b* (with $b \in H, b \in \mathrm{Re}v, b \notin D$) that are non-basic and have a non-negative reduced cost are stored in the set *J*. Then, backward reactions in *J* are fixed to zero, and a positive flux for their associated forward reactions is enabled, as shown in Eq. (10-11).

$$v_f = 0, 0 \le v_b \le v_b^{\max} \quad \forall (f,b) \in H, \forall (f,b) \in \mathrm{Re}v, \forall (f,b) \notin D, b \notin J \qquad (10)$$

$$v_b = 0, 0 \le v_f \le v_f^{\max} \quad \forall (f,b) \in H, \forall (f,b) \in \mathrm{Re}v, \forall (f,b) \notin D, b \in J \qquad (11)$$

Therefore, we solve the following optimization problem: Eq. (8) subject to Eq. (1)-(5), (7), (10)-(11). We repeat this process but starting with the reactions in the opposite direction, this is, switching *f* and *b* in Eq. (9)-(11). The whole procedure is repeated until no new reaction from *H* is added to the network. We refer to this procedure as **Iteration B** (see Figure A2).

Once Iteration B has ended, we may have reactions in *J* not included in *D*. However, some of them could possibly be included in the reconstruction. The reason for not having them included during Iteration B is that we should have reversed only a subset of them. Thus, the final step is to apply the procedure described in Iteration A for those reactions that remain included in *J* but not in *D*.

1.2 Reaction classification

Reactions are classified as highly, medium or lowly expressed using gene-protein-reaction rules and the gene expression classification [8] as mentioned in the main paper. These rules establish a relationship between the enzymes that catalyze each reaction and the genes that code for those enzymes. A reaction may be catalyzed by a single enzyme, different isozymes or a protein complex. Reactions having OR rules associated can be catalyzed by different isozymes, while those having AND rules involve protein complexes.

If a reaction is associated to a single gene, it is classified as *H*, *M* or *L* depending on the classification of the corresponding gene. If it involves an OR rule, it is classified as *H* if one of the genes is classified as *H*. On the contrary, it is classified as *L* if all the genes are classified as *L*. If a reaction involves an AND rule, it is classified as *H* if all the genes are classified as *H*, while as *L* if any of the genes is classified as *L*.

Those reactions for which no gene expression is available or that are not related to any gene (e.g. spontaneous reactions) are classified as medium expressed.

1.3 Gene Essentiality Analysis

A gene whose knock-out decreases the flux through the biomass reaction below 1e-6 is considered as essential. Based on empirical computational evidence, we found that this threshold has limited influence in the results.

2 SUPPLEMENTARY RESULTS

2.1 iMAT reconstruction coverage comparison

There are several proposals of reconstruction algorithms in the literature, like MBA [5], MIRAGE [9], GIMME [10], GIM^3E [11], INIT [12], iMAT [6], MADE [13]. Most of these algorithms rely on Mixed Integer Linear Programming (MILP) in order to select the active reactions for the contextualized reconstruction according to some predefined optimality criteria. Usually, each reaction is assigned a score as to how likely it is to be present in the reconstruction under consideration. This score can be obtained from genomics, transcriptomics, proteomics or other sources of data, or even from a combination of some or all of them. All the reconstruction methods have their place as some may be better suited for the integration of one type of data than others. Likewise, the results obtained from each one of them are not easily comparable, as each one aims for slightly different things.

In our case, our algorithm is closest (in the way it treats the inclusion of reactions) to iMAT [6]. The original optimization model proposed by iMAT for network reconstruction is as follows:

$$\max_{v,y^+,y^-}\left(\sum_{i\in R_H}\left(y_i^+ + y_i^-\right) + \sum_{i\in R_L} y_i^+\right) \quad (12)$$

subject to:

$$S\cdot v = 0 \quad (13)$$

$$v_{\min} \le v \le v_{\max} \quad (14)$$

$$v_i + y_i^+\left(v_{\min,i} - \varepsilon\right) \ge v_{\min,i}, i \in R_H \quad (15)$$

$$v_i + y_i^-\left(v_{\max,i} + \varepsilon\right) \le v_{\max,i}, i \in R_H \quad (16)$$

$$v_{\min,i}\left(1 - y_i^+\right) \le v_i \le v_{\max,i}\left(1 - y_i^+\right), i \in R_L \quad (17)$$

$$v \in R^m \quad (18)$$

$$y_i^+, y_i^- \in [0,1] \quad (19)$$

These optimization problem aims to strike a balance between the inclusion of *H* reactions and the exclusion of *L* reactions. It does not directly consider the inclusion of reactions that are not *H* or *L*. Our algorithm includes a term to control the inclusion of those reactions (*M* set) and promote compact solutions.

Aware of the possible existence of alternative solutions, iMAT proposes an iterative solution scheme to assign a confidence score for the inclusion or exclusion of each reaction. This step, however, is not compulsory for our task, and we will only solve the optimization problem once.

iMAT does not require a definition for the growth medium nor the specification of a biomass function, although they can be included into the formulation if desired [6]. When comparing iMAT to our algorithm, we will set the same medium conditions and ask for a minimum biomass production in order to have them in the same conditions. Another modification we will introduce to iMAT is in the definition of ε, which will be selected depending on the reaction bounds, as we do in our algorithm.

$$\max_{v,y^+,y^-}\left(\sum_{i\in R_H}\left(y_i^+ + y_i^-\right) + \sum_{i\in R_L} y_i^+\right) \quad (20)$$

subject to:

$$S\cdot v = 0 \quad (21)$$

$$v_{\min} \le v \le v_{\max} \quad (22)$$

$$v_{biomass} \ge v_{biomass}^* \quad (23)$$

$$v_i + y_i^+\left(v_{\min,i} - \varepsilon_{v_{\max},i}\right) \ge v_{\min,i}, i \in R_H \quad (24)$$

$$v_i + y_i^-\left(v_{\max,i} + \varepsilon_{v_{\min},i}\right) \le v_{\max,i}, i \in R_H \quad (25)$$

$$v_{\min,i}\left(1 - y_i^+\right) \le v_i \le v_{\max,i}\left(1 - y_i^+\right), i \in R_L \quad (26)$$

$$v \in R^m \quad (27)$$

$$y_i^+, y_i^- \in [0,1] \quad (28)$$

$$\varepsilon_{v_{\max},i} = \delta\cdot\left|v_{\max}\right| \quad (29)$$

$$\varepsilon_{v_{\min},i} = \delta\cdot\left|v_{\min}\right| \quad (30)$$

We select δ=0.10, the same value we use in our algorithm. When retrieving the solution, we consider as active any reaction with flux greater than 10^{-8}. If ε is lower than 10^{-8}, we set it to that quantity instead.

We use Cplex to solve the optimization problem. In addition, we exit the optimization process when the relative optimality gap is below 0.5% (gap between the relaxed problem solution and the best integer solution found), as closing the gap completely can be extremely memory and time consuming and adds little to the solution quality.

We used this implementation of iMAT and our algorithm to reconstruct cancer networks with gene expression obtained from the Cancer Cell Line Encyclopedia (CCLE) and Recon2 for the base network as in the main paper. The median time to obtain a network with iMAT was 57.15 seconds, while it was 2.14, 2.94 and 1.81 for schemas 1, 2 and 3 of our algorithm, respectively. This is clearly an improvement over the computation time needed to obtain a reconstruction.

In terms of how similar the reconstructions with each algorithm are, we can compare the percentage of *H* and *L* reactions included in each case. In Figure B, we plot the difference between the percentage of *L* reactions included with iMAT and the percentage

included with our algorithm using Schema 2 versus the difference between the percentage of *H* reactions included with iMAT and the percentage included with our algorithm. We chose Schema 2 because it is the one that behaves most similar to iMAT. It can be observed that the number of *L* reactions included is very similar and the number of *H* reactions included by our algorithm is somewhat lower. Overall, both methods obtain similar reconstructions in terms of the number of *H* and *L* reactions they include. Thus, we consider our algorithm a valid tool for the task at hand. In the case of the other schemas, for schema 3 our algorithm includes more *H* and *L* reactions than iMAT, and for schema 1 it includes less, as expected.

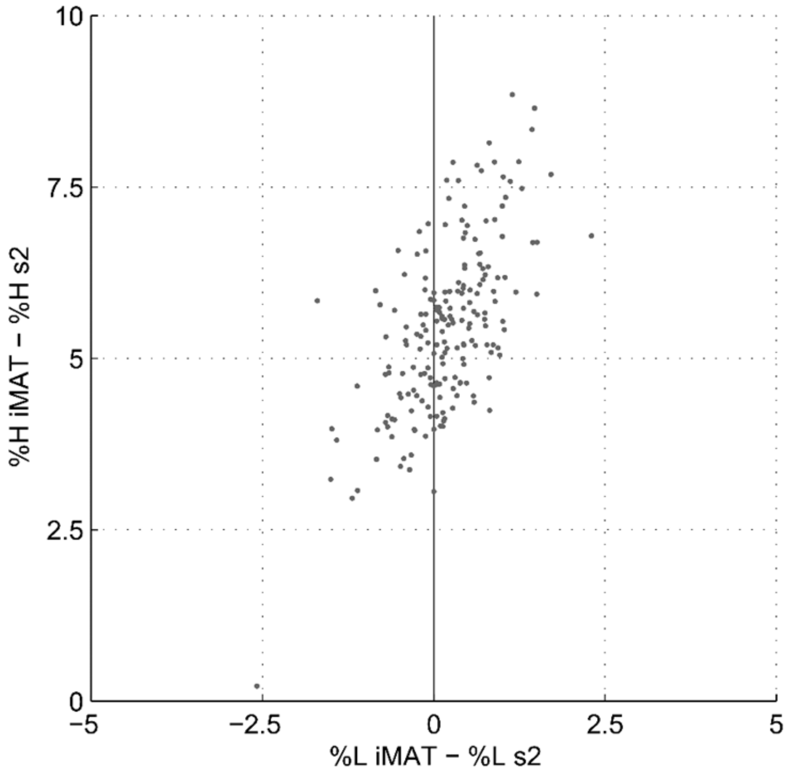

Figure B. Comparison between the percentage of *L* and *H* reactions included with iMAT or with our reconstruction algorithm with schema 2 in networks reconstructed starting form Recon 2. It can be observed that both include a similar percentage of *L* reactions and iMAT includes a slightly higher percentage of *L* reactions.

2.2 Model parameters and reconstruction

Here, we replicate the same analysis done in the main paper using Recon 1 instead of Recon2. The computation time is lower than in the case of Recon2, mainly because Recon1 is smaller than Recon2 (see Figure C).

Figure D also maintains the same trend observed in the main paper, where we have trade-off between reactions in *H* and in *L*, namely avoiding the inclusion of *L* reactions lowers the final count of included *H* reactions, while giving priority to *H* reactions increases the amount of *L* reactions included in the final model.

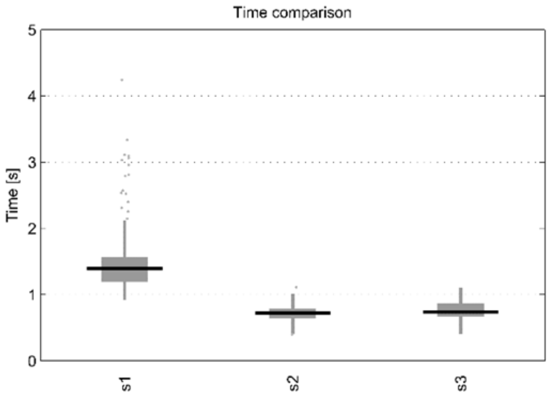

Figure C. Boxplot showing the computation times for reconstructed context-specific networks of selected cancer cell lines using our algorithm under schema 1, 2 and 3. The reference network used was Recon1.

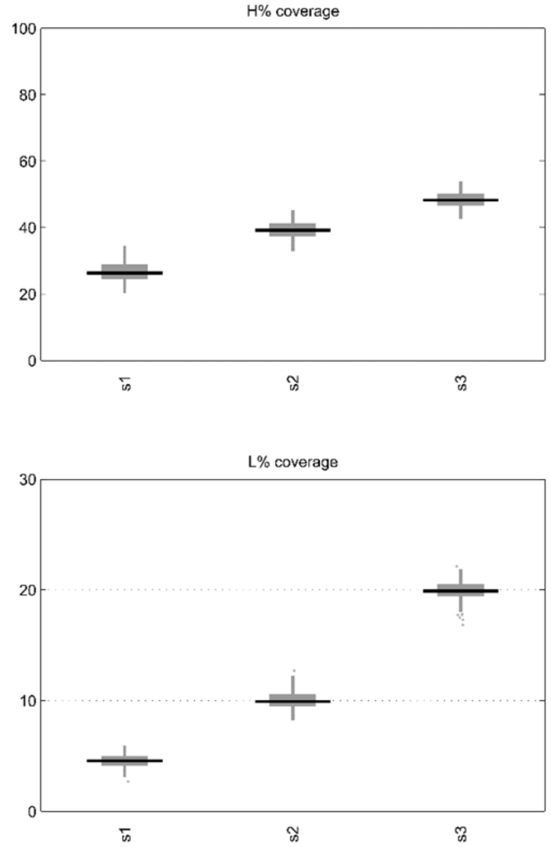

Figure D. Boxplots showing the percentage of *H* and *L* reactions included in the reconstructed context-specific networks of selected cancer cell lines using our algorithm under schema 1, 2 and 3. The reference network used was Recon1.

In addition to replicating the analysis using Recon1 instead of Recon2, we also substituted the values of some parameters one by one in order to test the robustness of our approach. In the results presented in the main paper, we fixed $\alpha=10^3$, $v^*_{biomass}=0.01\cdot v^{\max}_{biomass}$ and $\delta=0.10$. Here, we substituted the value of $\alpha=10^3$ by $\alpha=10^2$, the value of $v^*_{biomass}=0.01\cdot v^{\max}_{biomass}$ by $v^*_{biomass}=0.10\cdot v^{\max}_{biomass}$ and the value of $\delta=0.10$ by $\delta=0.01$. Whenever we changed one of these parameters, we left the others with the values used in the main paper. Furthermore, we also tested a very general medium in substitution of the RPMI1640 medium used in the main paper. This general medium was composed by those metabolites with an annotated input reaction in the reference network. We provide the mean computation times and *H* and *L* coverage in Table A, finding a robust solution for each different schema. As partially expected, the major difference was found in the case of the usage of a general growth medium.

Table A. Average computation time, percentage of included *H* reactions and percentage of included *L* reactions under different parameter settings.

Setup	Schema	Time [s]	H%	L%
$\alpha=10^2$	1	2.14	34.73	1.84
	2	2.99	56.17	8.36
	3	1.80	69.04	20.47
$\delta=0.01$	1	2.57	41.68	1.65
	2	3.51	57.54	8.00
	3	2.91	70.11	18.53
$v^*_{biomass}=0.10\cdot v^{\max}_{biomass}$	1	2.26	34.63	1.65
	2	3.06	56.22	8.36
	3	1.80	69.06	21.20
General growth medium	1	2.22	41.30	0.42
	2	3.16	61.55	7.98
	3	2.47	75.20	23.70

2.3 Gene essentiality analysis

The selected CCLE cell lines include samples from 20 different cancer types: bladder, bone sarcoma, breast, colon, endometrial, esophageal, glioblastoma, gastric, leukemia, liver, lung mesothelioma, lung NSCLC, lung SCLC, melanoma, multiple myeloma, ovarian, pancreas, prostate, renal cell carcinoma and soft tissue sarcoma. As stated in the main text, we found a small number of genes that appear exclusively in samples from one cancer type. With the settings used in the main paper, schema 1 gives in total 22 genes that appear only in one cancer type, schema 2 gives 21 and schema 3 gives 6. For the parameter settings used in Table A, a similar conclusion can be achieved (see Table B). Figure E, Figure F, Figure G and Figure H are analogous to Figure 3 in the main text, but focusing on the GBM samples present in CCLE, U251 samples from GEO, U87 samples from GEO and A549 samples from GEO, respectively. See S1 Table for a list of the samples used.

Table B. Number of genes that appear as essential in samples from one single cancer type.

Setup	Schema	Number of genes exclusive of one cancer type
$\alpha=10^2$	1	20
	2	34
	3	11
$\delta=0.01$	1	30
	2	20
	3	14
$v^*_{biomass}=0.10\cdot v^{\max}_{biomass}$	1	29
	2	24
	3	4
General growth medium	1	19
	2	19
	3	18
Recon 1	1	21
	2	17
	3	7

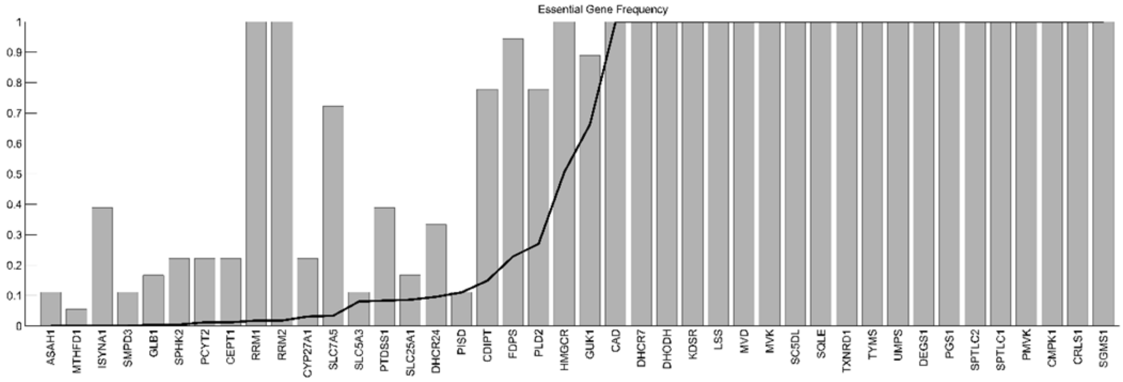

Figure E. Essential gene frequency in networks reconstructed from GBM samples in CCLE with matched Achilles experiments using our algorithm with schema 3 and Recon2 as the reference network. The horizontal axis contains the ENTREZ Symbols of the obtained essential genes. The height of the bars indicates the fraction of samples in which the gene appears as essential. The height of the black line indicates the fraction of randomly reconstructed network in which the corresponding gene appears as essential.

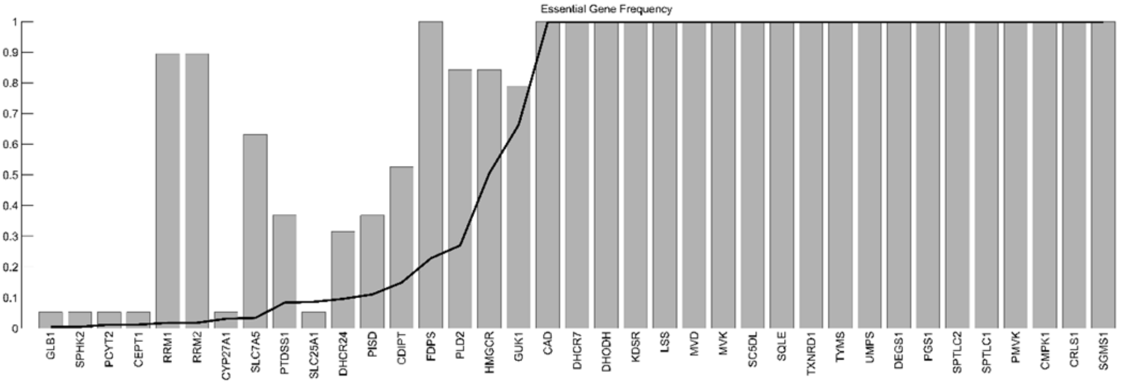

Figure F. Essential gene frequency in networks reconstructed from U251 samples using our algorithm with schema 3 and Recon2 as the reference network. The horizontal axis contains the ENTREZ Symbols of the obtained essential genes. The height of the bars indicates the fraction of samples in which the gene appears as essential. The height of the black line indicates the fraction of randomly reconstructed network in which the corresponding gene appears as essential.

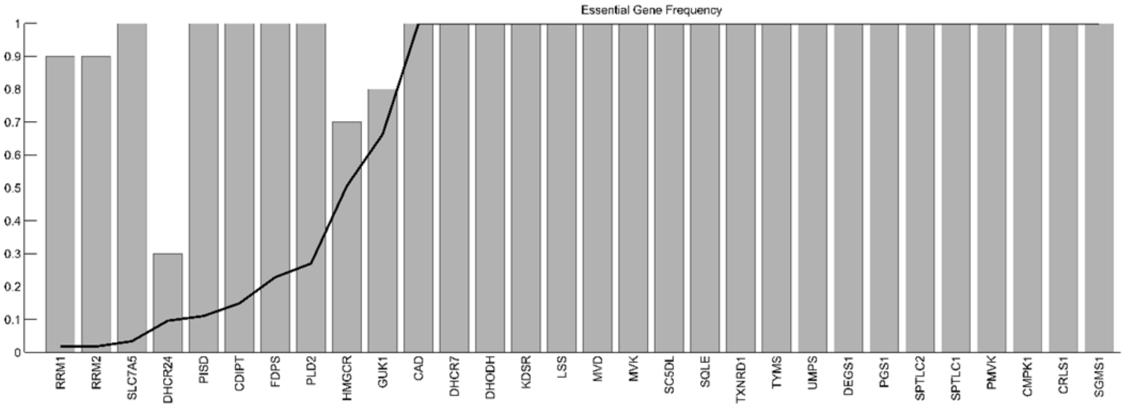

Figure G. Essential gene frequency in networks reconstructed from U87 samples using our algorithm with schema 3 and Recon2 as the reference network. The horizontal axis contains the ENTREZ Symbols of the obtained essential genes. The height of the bars indicates the fraction of samples in which the gene appears as essential. The height of the black line indicates the fraction of randomly reconstructed network in which the corresponding gene appears as essential.

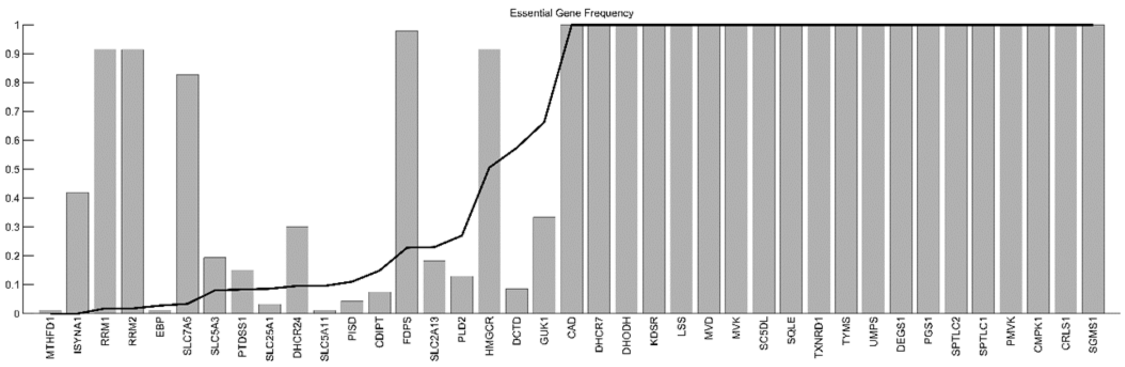

Figure H. Essential gene frequency in networks reconstructed from A549 samples using our algorithm with schema 3 and Recon2 as the reference network. The horizontal axis contains the ENTREZ Symbols of the obtained essential genes. The height of the bars indicates the fraction of samples in which the gene appears as essential. The height of the black line indicates the fraction of randomly reconstructed network in which the corresponding gene appears as essential.

2.4 Comparison to high-throughput gene silencing experiments

We replicate here the same analysis done in the main paper for comparing the obtained set of essential genes with the reported scores for Project Achilles using Recon1, instead of Recon2. The results are very similar to the ones obtained in the main paper. For other parameter settings, the median values of the KS test p-values and the percentage of essential genes with a negative score can be found in Table C.

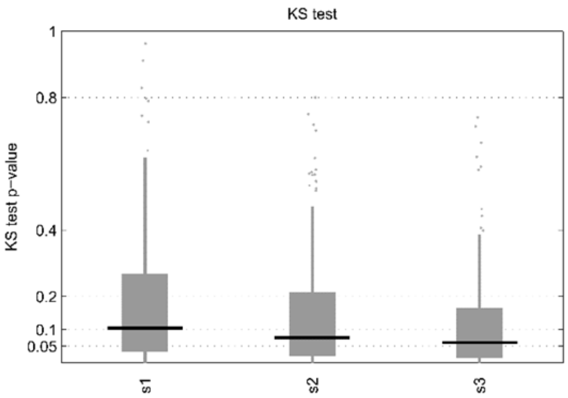

Figure I. KS test p-value of essential genes obtained from the networks reconstructed from CCLE samples with matched Achilles experiment using our algorithm under schema 1, 2 and 3. The base network is Recon1.

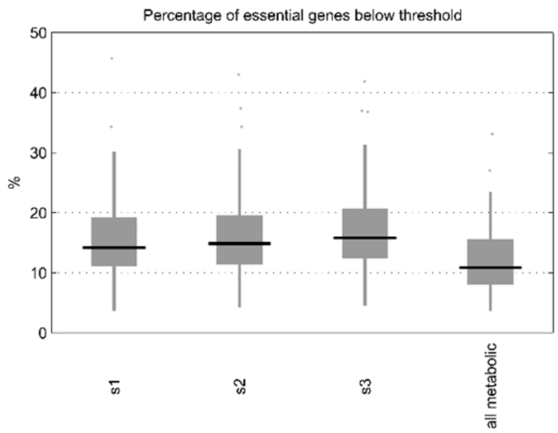

Figure J. Percentage of essential genes obtained from the reconstructed context-specific networks of selected cancer cell lines using our algorithm under schema 1, 2 and 3 that have a negative score, indicating a higher probability of being essential. The base network is Recon1. The last boxplot indicates this percentage when all the metabolic genes are taken into account.

Table C. Median KS test p-value and percentage of essential genes with negative score under different parameter settings.

Setup	Schema	KS test p-value	Percentage of essential genes below threshold
α =10^2	1	0.1508	0.1403
	2	0.3852	0.1400
	3	0.1373	0.1852
δ =0.01	1	0.1050	0.1505
	2	0.3000	0.1530
	3	0.3276	0.1500
$v^*_{biomass}$ =0.10· $v^{\max}_{biomass}$	1	0.1275	0.1509
	2	0.3694	0.1429
	3	0.1068	0.2034
General medium	1	0.4331	0.1429
	2	0.4863	0.1429
	3	0.3638	0.2000

As mentioned in the main text, we tried to leverage the information on the frequency of appearance of the computationally obtained essential genes in the randomly reconstructed networks and networks reconstructed from samples of a same type of cell line with respect to the experimental essentiality data, but we did not extract any conclusive results, most likely due to small sample size. However, when we merged the results for the different CCLE samples, in the case of the ones obtained from the reconstructions with schema 3, we observed that when we focused only on essential genes with a frequency of appearance in the randomly reconstructed networks lower than a given value, as we decreased this value, the percentage of calculated essential genes with a negative Achilles-based score underwent a moderate increase (Figure K and Figure L).

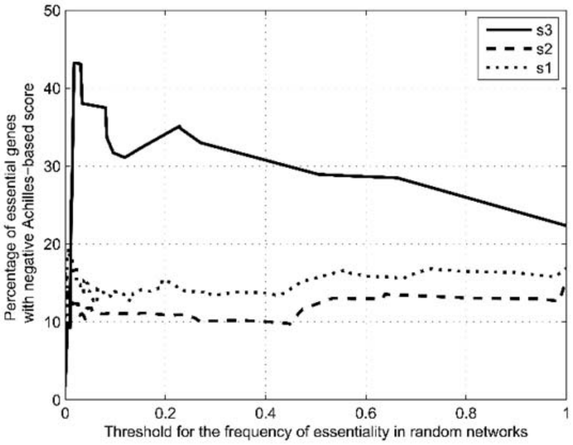

Figure K. Percentage of computationally obtained essential genes satisfying Achilles-based threshold of essentiality as a function of their frequency of appearance in randomly reconstructed networks. For a given threshold value (x-axis), we took all the calculated essential genes that appeared with a lower frequency than that threshold in the networks reconstructed from random expression, and calculated the percentage of genes associated to a negative Achilles-based score (y-axis). The networks from which the essential genes were calculated were reconstructed from the CCLE samples using Recon2 as the base network and the default set of parameters used in the main text.

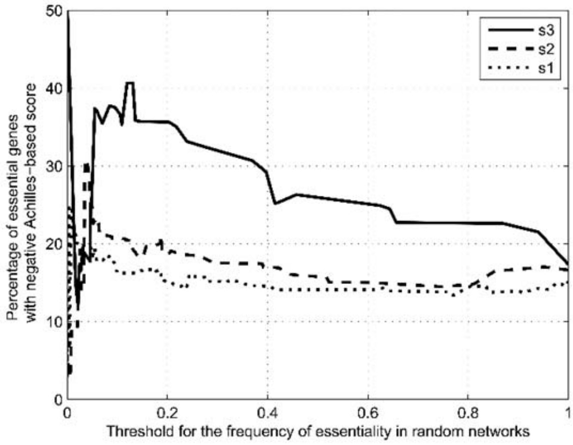

Figure L. Percentage of computationally obtained essential genes satisfying Achilles-based threshold of essentiality as a function of their frequency of appearance in randomly reconstructed networks. For a given threshold value (x-axis), we took all the calculated essential genes that appeared with a lower frequency than that threshold in the networks reconstructed from random expression, and calculated the percentage of genes associated to a negative Achilles-based score (y-axis). The networks from which the essential genes were calculated were reconstructed from the CCLE samples using Recon1 as the base network and the default set of parameters used in the main text.

2.5 Random alterations of the biomass reaction

We decided to change the biomass reaction in a random fashion to observe how the KS test p-values could change if the biomass reaction was different. On a first test, we randomly varied the coefficients of the metabolites that participated in the biomass reaction up to a ±50% (we excluded from these modifications metabolites *h2o[c]*, *atp[c]*, *adp[c]*, *h[c]* and *pi[c]*, linked to ATP maintenance). Figure M shows the results of 10 experiments, each one with a different random alteration on the biomass reaction. As can be observed, the results are extremely similar, which tells us that the specific values of the coefficients in the biomass reaction are not of critical importance in this case.

Next, we decided to randomly set to zero some of the coefficients of the metabolites participating in the biomass reaction. In each experiment, we selected a random number between 0.05 and 0.50 and used it as the probability with which we would set to zero the coefficients of the metabolites participating in the biomass reaction (again, we excluded from these modifications metabolites *h2o[c]*, *atp[c]*, *adp[c]*, *h[c]* and *pi[c]*, linked to ATP maintenance). Figure N shows the results of 10 experiments following this strategy, where there are cases with worsened results. This shows that changing the composition of the biomass does indeed have a strong effect on the results. In this case, dropping some components clearly worsened the results, while dropping others did not have any appreciable consequence. In order to improve the results, it is likely that we will need to include new metabolites into the composition of the biomass reaction equation. Note here that a similar result was found when we determined the proportion of essential genes satisfying the Achilles-based threshold of essentiality, which reinforces the relevance of the selection of the biomass equation.

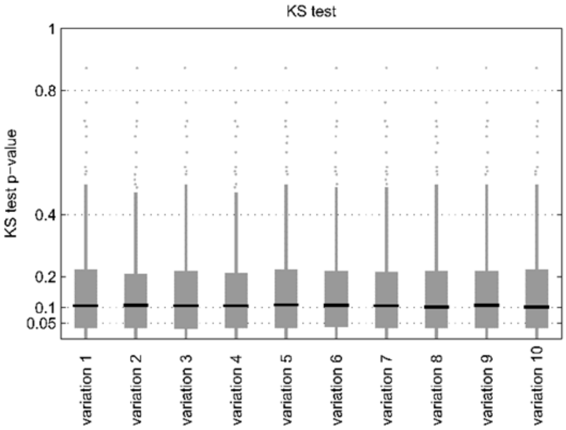

Figure M. KS test p-value results after altering some of the coefficients of the metabolites participating in the biomass reaction up to a ±50%. The experiment consisted of 10 different random variations. The base network used was Recon 2 and the networks were reconstructed from the CCLE samples with matched Achilles experiments using our algorithm with schema 3 and the default parameters used in the main paper.

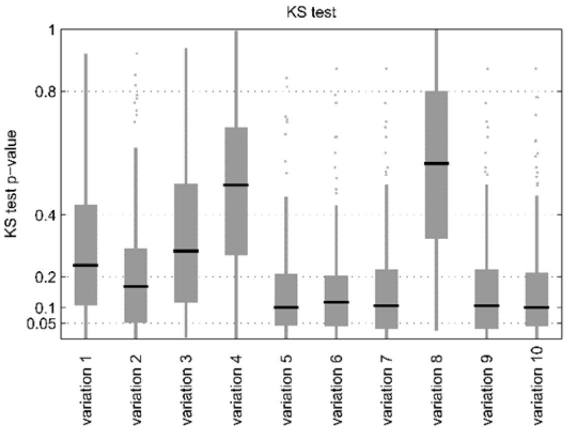

Figure N. KS test p-value results after setting some of the coefficients of the metabolites participating in the biomass reaction to zero. The experiment consisted of 10 different random variations, where, in each variation, the probability of setting the coefficients to zero was randomly chosen. The base network used was Recon 2 and the networks were reconstructed from the CCLE samples with matched Achilles experiments using our algorithm with schema 3 and the default parameters used in the main paper.

REFERENCES

1. Palsson BØ. Systems biology : properties of reconstructed networks. New York, NY, USA: Cambridge University Press; 2006.

2. Figueiredo LF de, Podhorski A, Rubio A, Kaleta C, Beasley JE, Schuster S, et al. Computing the shortest elementary flux modes in genome-scale metabolic networks. Bioinformatics. 2009;25: 3158–3165. doi:10.1093/bioinformatics/btp564

3. Gudmundsson S, Thiele I. Computationally efficient flux variability analysis. BMC Bioinformatics. 2010;11: 489. doi:10.1186/1471-2105-11-489

4. Vlassis N, Pacheco MP, Sauter T. Fast Reconstruction of Compact Context-Specific Metabolic Network Models. PLoS Comput Biol. 2014;10: e1003424. doi:10.1371/journal.pcbi.1003424

5. Jerby L, Shlomi T, Ruppin E. Computational reconstruction of tissue-specific metabolic models: application to human liver metabolism. Mol Syst Biol. 2010;6.

6. Shlomi T, Cabili MN, Herrgard MJ, Palsson BO, Ruppin E. Network-based prediction of human tissue-specific metabolism. Nat Biotech. 2008;26: 1003–1010.

7. Vanderbei R. Linear Programming: Foundations and Extensions. No. 4 in International series in operations research & management. Boston, Massachusetts: Kluwer Academic Publishers; 1996.

8. Rossell S, Huynen MA, Notebaart RA. Inferring Metabolic States in Uncharacterized Environments Using Gene-Expression Measurements. PLoS Comput Biol. 2013;9: e1002988. doi:10.1371/journal.pcbi.1002988

9. Vitkin E, Shlomi T. MIRAGE: a functional genomics-based approach for metabolic network model reconstruction and its application to cyanobacteria networks. Genome Biology. 2012;13: R111. doi:10.1186/gb-2012-13-11-r111

10. Becker SA, Palsson BO. Context-Specific Metabolic Networks Are Consistent with Experiments. PLoS Comput Biol. 2008;4: e1000082. doi:10.1371/journal.pcbi.1000082

11. Schmidt BJ, Ebrahim A, Metz TO, Adkins JN, Palsson BØ, Hyduke DR. GIM3E: condition-specific models of cellular metabolism developed from metabolomics and expression data. Bioinformatics. 2013;29: 2900–2908. doi:10.1093/bioinformatics/btt493

12. Agren R, Bordel S, Mardinoglu A, Pornputtapong N, Nookaew I, Nielsen J. Reconstruction of Genome-Scale Active Metabolic Networks for 69 Human Cell Types and 16 Cancer Types Using INIT. PLoS Comput Biol. 2012;8: e1002518. doi:10.1371/journal.pcbi.1002518

13. Jensen PA, Papin JA. Functional integration of a metabolic network model and expression data without arbitrary thresholding. Bioinformatics. 2011;27: 541–547. doi:10.1093/bioinformatics/btq702
